# Identification of the Unique Clinical and Genetic Features of Chinese Lung Cancer Patients With *EGFR* Germline Mutations in a Large-Scale Retrospective Study

**DOI:** 10.3389/fonc.2021.774156

**Published:** 2021-11-16

**Authors:** Xinqing Lin, Muyun Peng, Quanfang Chen, Mingming Yuan, Rongrong Chen, Haiyi Deng, Jiaxi Deng, Ouqi Liu, Yuqing Weng, Mingjiu Chen, Chengzhi Zhou

**Affiliations:** ^1^ State Key Laboratory of Respiratory Disease, National Clinical Research Centre for Respiratory Disease, Guangzhou Institute of Respiratory Health, First Affiliated Hospital, Guangzhou Medical University, Guangzhou, China; ^2^ Department of Thoracic Surgery, Hunan Key Laboratory of Early Diagnosis and Precise Treatment of Lung Cancer, The Second Xiangya Hospital of Central South University, Changsha, China; ^3^ Department of Respiratory Medicine, First Affiliated Hospital of Guangxi Medical University, Nanning, China; ^4^ Medical Department, Geneplus-Beijing, Beijing, China; ^5^ Department of Respiratory and Critical Medicine, Zhuhai People’s Hospital (Zhuhai Hospital Affiliated with Jinan University), Zhuhai, China

**Keywords:** genetic features, EGFR, treatment, Chinese lung cancer patient, germline mutations

## Abstract

**Background:**

Epidemiological surveys have suggested that lung cancer has inherited susceptibility and shows familial aggregation. However, the distribution and prevalence of epidermal growth factor receptor (*EGFR*) germline variants and their roles in lung cancer genetic predisposition in Chinese population remain to be elucidated.

**Methods:**

In this study, *EGFR* germline and somatic variants were retrospectively reviewed from the next-generation sequencing results of 31,906 patients with lung cancer. Clinical information was also collected for patients with confirmed EGFR germline mutations.

**Results:**

A total of 22 germline *EGFR* variants were identified in 64 patients with lung cancer, accounting for 0.2% of the total cases studied. Five patients were diagnosed as multiple primary carcinomas. Family history was documented in 31.3% (20/64) of patients, 55% of which were diagnosed as lung cancer. G863D was the most frequent *EGFR* germline mutation, followed by P848L, D1014N, and K757R. Somatic *EGFR*-sensitive mutations were identified in 51.6% of patients with germline *EGFR* mutations. The proportion of L858R mutation, exon 19 deletion, and rare sensitive mutation was 50%, 17.6%, and 32.4%, respectively. D1014N and T790M mutations were common in young patients. The family members of patients with P848L, R776H, V769M, and V774M mutations were more commonly diagnosed with cancers. A total of 19 patients were confirmed to have received EGFR tyrosine kinase inhibitors (TKIs), but the response to EGFR-TKIs differed among patients with different *EGFR* mutations.

**Conclusion:**

Chinese patients with lung cancer harbored unique and dispersive *EGFR* germline mutations and showed unique clinical and genetic characteristics, with varied response patterns to EGFR-TKI treatment.

## Introduction

Lung cancer is the most common and lethal malignancy in most countries. China reported 733,300 new cases and 610,200 lung cancer deaths in 2015 (1). Tobacco smoking is the greatest risk factor for lung cancer development, with up to 80% of cases attributed to smoking (2). Recently, additional risk factors, including exposure to radon, occupational hazards, biomass fuel, and infectious diseases have been identified as additional risk factors in the carcinogenesis of lung cancer (2).

Epidemiological surveys have further suggested that lung cancer has inherited susceptibility and show familial aggregation (3–6). That is, genetic factors, such as high-frequency single nucleotide polymorphisms with low penetrance and low-frequency pathogenic germline variants with high penetrance, have been confirmed to be related to lung cancer predisposition (7–10). Multiple genome-wide association studies confirmed *CHRNA5*, *TERT*, *BAT2*, and *FKBPL* as candidate genes associated with lung cancer risk (6, 7). The investigation of pathogenic germline variants mainly focused on epidermal growth factor receptor (*EGFR*) and other genes commonly related to hereditary tumor syndromes, including *ATM*, *TP53*, and *BRCA2* (11). There are four well-documented germline mutations in *EGFR*, including T790M, V843I, R776X, and P848L (12). *EGFR* T790M is the most frequent mutation in Western countries, with a 0.54% frequency in nonsmokers and 0.34% in patients with nonsquamous nonsmall cell lung cancer (NSCLC) (13, 14). However, the frequency of EGFR T790M germline mutation in Chinese lung cancer patients was 0.0078%, suggesting a distinct germline mutation spectrum among different ethnicities (15). Therefore, the distribution and prevalence of *EGFR* germline variants and their roles in lung cancer genetic predisposition in Chinese population remain to be elucidated.

In this study, EGFR germline and somatic variants were retrospectively reviewed in 31,906 patients with lung cancer whose tumor tissues or peripheral blood samples were collected to perform a matched tumor-normal next-generation sequencing of 1,021 cancer-related genes. Clinical information was also collected for each patient identified with EGFR germline mutations for comparison.

## Methods

### Patients and Samples

This study recruited a total of 31,906 Chinese patients with lung cancer who underwent matched tumor-normal next-generation sequencing at Geneplus-Beijing (Beijing, China) from April 2015 to March 2021. Tumor tissues (including formalin-fix paraffin-embedded, frozen, and needle biopsy samples), peripheral blood samples, or effusion samples were obtained from each participant. This study was approved by the Ethics Committee of the First Affiliated Hospital, Guangzhou Medical University (Guangzhou, China) (Approval No. 2020-140). All procedures were conducted in accordance with the Declaration of Helsinki and written informed consent for mutational analysis of genomic DNA (gDNA) and circulating free DNA (cfDNA) was obtained from all participants.

### Sample Processing and DNA Extraction

Peripheral blood samples were collected in Streck tubes (Streck, Omaha, NE, USA) and centrifuged within 72 h to separate the plasma from the peripheral blood cells. To detect germline and somatic mutations, gDNA was extracted from the peripheral blood cells and fresh tumor tissues using a QIAamp DNA Blood mini kit (Qiagen, Hilden, Germany). Formalin-fixed, paraffin-embedded (FFPE) DNA was isolated using Maxwell^®^ 16 FFPE Plus LEV DNA purification kit (Qiagen, Hilden, Germany). QIAamp Circulating Nucleic Acid Kit (Qiagen, Hilden, Germany) was used to extract cfDNA from liquid biopsies. DNA extractions were performed according to the manufacturer’s instructions. The DNA concentration was measured using a Qubit fluorometer and Qubit dsDNA HS (high sensitivity) assay kit (Invitrogen, Carlsbad, CA, USA).

### Library Preparation, Target Capture, and Sequencing

Sequencing libraries were prepared from ctDNA using KAPA DNA Library preparation kits (Kapa Biosystems, Wilmington, MA, USA), and genomic DNA sequencing libraries were prepared using Illumina’s TruSeq DNA Library preparation kits (Illumina, San Diego, CA, USA). Libraries were hybridized to custom-designed biotinylated oligonucleotide probes (Roche NimbleGen, Madison, WI, USA) targeting cancer-related genes ranging from 16 to 1,021, including but not limited to all driver mutations in lung cancer (*EGFR*, *ALK*, *ROS1*, *RET*, *KRAS*, *NRAS*, *TP53*, *BRAF*, *ERBB2*, and *MET*).

### Sequencing Data Analysis

Terminal adaptor sequences were removed from the raw sequencing data. Subsequently, reads with more than 50% low-quality bases, or more than 50% undefined bases, were discarded. The remaining reads were mapped to the reference human genome (hg19) using the Burrows-Wheel Aligner (BWA). Somatic variants, including single nucleotide variants (SNVs), small insertions and deletions (InDels), copy number alterations (CNAs), and structural variants were assessed. MuTect2 (version 1.1.4) and NChot2 were employed to identify somatic SNVs, while GATK was used to identify small insertions and deletions (indels). CNAs were identified using Contra (v.2.0.8). Structural variants (SVs) were identified using NCsv (an in-house tool). The candidate variants were all manually verified using the Integrative Genomics Viewer.

### Clinical and Genetic Data Analysis

All nonsynonymous variants in the coding region of *EGFR* gene were screened, and variants with frequencies greater than 0.01 in general populations were excluded. A variant was included in the final analysis cohort only when: (i) it was reported to be associated with targeted therapy; (ii) previously documented as a germline variant; or (iii) reported as functional. Clinical characteristics such as age at diagnosis, family history, and treatment history were collected for each patient in the final analysis cohort.

### Statistical Analysis

The difference in age at diagnosis between different groups was evaluated using a two-tailed unpaired Mann-Whitney *U* test. Fisher’s exact test was utilized to assess the differences in other demographic characteristics. Statistical significance was determined at *p* < 0.05.

## Results

### Patient Characteristics

In the final analysis cohort, a total of 22 germline *EGFR* variants were identified in 64 patients with lung cancer. The prevalence of *EGFR* germline mutation in Chinese patients with lung cancer was 0.2% (64/31,906), which was higher than that found in a previous study (15). The baseline characteristics are summarized in **Table 1**. The median age at diagnosis was 61.5 years (range: 44–88 years). Regarding the diagnosis, most patients were classified as adenocarcinoma (73.4%, 47/64), five patients with multiple primary carcinomas, including three patients with double primary lung adenocarcinomas, one patient with mucoepidermoid carcinoma and adenocarcinoma of the lung, and one patient with lung adenocarcinoma and liver cancer. More than half the patients (53.1%, 34/64) were ever smokers, and family history was documented for approximately one-third of the patients (31.3%, 20/64). Among those whose cancer family history was obtained, lung cancer was the most recurrent type among the family members of 55% (11/20) patients.

**Table 1:** Clinical characteristics of patients in the final analysis cohort.

Characteristics	Patients (*n* = 64)
Age at diagnosis^a^ (years)	
Median	61.5
Range	44–88
Gender [No. (%)]	
Female	29 (45.3%)
Male	34 (53.1%)
NA	1 (1.6%)
Histologic subtype [No. (%)]	
Adenocarcinoma	47 (73.4%)
Squamous cell carcinoma	2 (3.1%)
NA	10 (15.6%)
Multiple primary carcinomas	5 (7.8%)
Smoking history [No. (%)]	
Yes	34 (53.1%)
No	22 (34.3%)
NA	8 (12.5%)
Family history [No. (%)]	
Yes	20 (31.3%)
No	37 (57.8%)
NA	7 (10.9%)

a Eight patients did not have diagnostic age information (missing data).

NA, not available.

### *EGFR* Germline and Somatic Mutations

**Figure 1:**
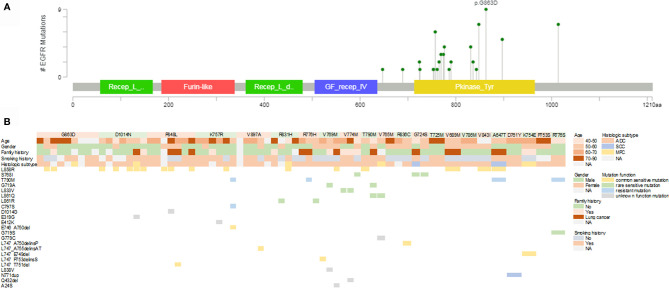
Mutational landscape of germline and somatic *EGFR* gene mutations. **(A)** Lollipop plot of the distribution of *EGFR* germline mutations. **(B)** Mutational profiles of patients with germline *EGFR* mutations. EGFR, epidermal growth factor receptor; ADC, adenocarcinoma; SCC, squamous cell carcinoma; MPC, multiple primary carcinoma; NA, not available.

In our cohort, the mutation spectrum of *EGFR* germline mutations was considerably different from another study evaluating Chinese cancer patients (**Figure 1A**) (15). G863D, identified in nine of our patients (14.1%), was the most frequent *EGFR* germline mutation, followed by P848L (10.9%), D1014N (10.9%), K757R (9.4%), V897A (7.8%), and R831H (6.3%). *EGFR*-T790M, the dominant *EGFR* germline mutation in Western countries, was only present in two cases in our cohort. Most mutations (86.4%, 19/22) occurred within the tyrosine kinase domain, except for A647T (one case), V689M (one case), and D1014N (seven cases).

A total of 46 *EGFR* somatic mutations were concurrently identified in 36 patients with *EGFR* germline mutations (**Figure 1B**). *EGFR*-sensitive mutations were identified in 51.6% (33/64) of patients with germline *EGFR* mutations. *EGFR* L858R was the most common mutation, with a detection rate of 26.6% (17/64). The distribution of deletion or deletion-insertion mutations in *EGFR* exon 19 was dispersive, accounting for 9.4% of the cases (6/64). Rare sensitive mutations, including S768I, G719A, L861Q/R, L833V, and G719S were found in 17.2% of patients (11/64). *EGFR*-resistant mutations were also identified in six cases, including three cases with T790M mutation, one with T790M and C797S mutations, and one with N771dup mutation.

### Clinical and Genetic Feature Comparison Among Patients With Different Germline Mutations

**Figure 2:**
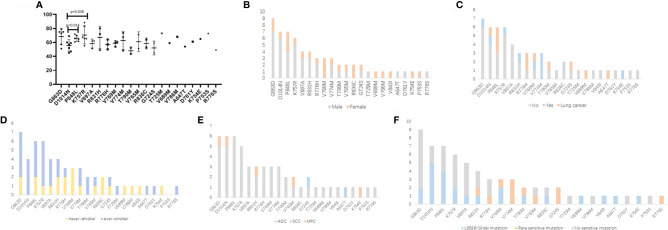
Comparison of clinical and genetic features among patients with different *EGFR* germline mutations. **(A)** Age at diagnosis (years). **(B)** Gender. **(C)** Cancer family history. **(D)** Smoking history. **(E)** Histologic subtype. **(F)** Somatic *EGFR-*sensitive mutations. ADC, adenocarcinoma; SCC, squamous cell carcinoma; MPC, multiple primary carcinoma.

We also investigated the possible differences in clinical and genetic characteristics among patients harboring different germline mutations. Compared with the age of patients with P848L or K757R germline mutation, that at diagnosis among patients with D1014N was significantly lower (median: 57 years for D1014N, 65.5 years for P848L, 66 years for K757R, *p* = 0.014 and 0.046, respectively). No significant differences were observed when comparing other groups (**Figure 2A**). Owing to the small number of patients in certain germline mutation groups, only those with ≥3 patients were included in the comparative analysis. More than three-quarters of patients with G863D, D1014N, and K757R were males. Whereas, all the patients with V769M (*n* = 3) and R836C (*n* = 2) were females. For the former, the differences were statistically significant (*p* = 0.045, 0.033, and 0.048, respectively) (**Figure 2B**). More than half of the patients with P848L, R776H, V769M, and V774M had cancer family history (including lung cancer). In addition, the percentage of lung cancer family history was higher than 50% in patients with P848L, R776H, and V769M (**Figure 2C**). All the patients with V769M mutation were never smokers, which was significantly different from patients with D1014N and K757R mutation (*p* = 0.048 and 0.029, respectively) (**Figure 2D**). Majority of patients were diagnosed as adenocarcinoma, with other subtypes dispersedly distributed across several groups. Multiple primary carcinomas were found in patients with G863D, D1014N, R831H, V765M, and K754E (**Figure 2E**). More than half of the patients with D1014N, P848L, and V769M harbored somatic exon 19 deletion or L858R mutations. Rare *EGFR*-sensitive mutations were frequently found in patients with V769M and V774M, but not in patients with G863D, D1014N, P848L, K757R, and V897A (**Figure 2F**).

### Patient Response to EGFR-TKIs

EGFR tyrosine kinase inhibitors (TKIs) were administered to patients with or without somatic *EGFR*-sensitive mutations. A total of 19 patients were confirmed to receive EGFR-TKIs; the survival information from their medical records is summarized in **Table 2**. Among patients with somatic L858R mutation (P1-P8), those with P848L (P4), V769M (P6), and K757R (P5) received the short duration of treatment (DOT) with EGFR-TKIs (2, 3, and 6 months, respectively). Only one patient with solely germline P848L mutation did not respond to EGFR-TKIs (P18). However, one patient with germline P848L mutation and somatic exon 19 deletion mutation had a DOT of 10 months (P9). Different from P5, one patient with germline K757R mutation and somatic exon 19 deletion mutation had a durable response to gefitinib and osimertinib (P10). A similar finding was observed in patients with V769M mutation, which showed that one patient with germline V769M mutation and somatic exon 19 deletion mutation had a DOT of 17 months for gefitinib (P11). Patients with germline D1014N (P1 and P2)/V843I (P8) mutation and somatic L858R mutation also responded well to EGFR-TKIs, with a DOT longer than 1 year. Patients with exon 19 deletion somatic mutation and R836C (P12)/K754E (P13) had a modest DOT with EGFR-TKIs. Germline T790M mutations were identified in two patients (P7 with L858R somatic mutation and P15 with somatic L861Q and G719A mutations). P7 showed a modest DOT for icotinib combined with two cycles of chemotherapy, but a durable DOT for the osimertinib group. The DOT for icotinib for P15 was 15 months. One patient with somatic primary T790M mutation and germline R776H had a DOT of 5 months for osimertinib (P17). In addition, patients with somatic S768I and germline V774M mutations (P14) and germline R831H were sensitive to EGFR-TKIs.

**Table 2:** The clinical and genetic features and patients’ response to EGFR-TKIs in patients with germline EGFR mutations.

Patient No.	Gender	Age at diagnosis	Smoking history	Family history	Histologic subtype	Stage	Germline mutation	Somatic mutations	EGFR-TKIs treatment (progression or not; DOT)
1	Male	52	NA	No	LADC	IV	D1014N	L858R	Gefitinib (yes, 16 m)
									Erlotinib (yes, 2 m)
2	Male	57	Yes	Mother/sister/brother, LC	LADC; Liver cancer	IV	D1014N	L858R	Gefitinib (combined with CT and sorafinib) (no, 14 m)
3	Female	70	No	Brother, LC; mother, NA	LADC	IV	P848L	L858R	Gefitinib (no, 2 m)
4	Male	72	Yes	Brother, liver cancer; sister, BC; brother, LC	LADC	IV	P848L	L858R	EGFR-TKI (yes, 2 m)
5	Male	83	No	No	LADC	IV	K757R	L858R	Icotinib (yes, 6 m)
6	Female	61	No	Mother, LADC	LADC	NA	V769M	L858R	Gefitinib (yes, 3 m)
7	Male	52	Yes	No	LADC	IV	T790M	L858R	Icotinib (combined with CT) (yes, 7 m)
									Osimertinib (yes, 18 m)
8	Female	NA	No	No	LADC	IV	V843I	L858R	Gefitinib (yes, 17 m)
									Osimertinib (yes, 2 m)
9	Female	66	No	Father/brother, LC	LADC	IV	P848L	L747_T751del	Gefitinib (yes, 10 m)
									Icotinib (NA, 8 m)
10	Male	55	Yes	No	LADC	IV	K757R	C797S, E746_A750del, T790M	Gefitinib (yes, 16 m)
									Osimertinib (yes, 25 m)
11	Female	61	No	No	LADC	IV	V769M	L747_P753delinsS	Gefitinib (yes, 17 m)
									Osimertinib (NA, NA)
12	Female	53	No	No	LADC	IV	R836C	L747_A750delinsP	EGFR-TKI (yes, 8 m)
13	Female	65	No	Mother, EC	LADC; LMC	NA	K754E	L747_T751del	Icotinib (no, 9 m)
14	Female	48	No	Grandmother, EC; father, RC	LADC	NA	V774M	S768I	Afatinib (combined with CT) (no, 20 m)
15	Female	44	Yes	Brother/sister, LC	LADC	NA	T790M	L861Q, G719A	Icotinib (yes, 15 m)
16	Male	49	Yes	No	LADC	IV	R776S	G719S, T790M (PCR)	Osimertinib (NA, 3 m)
17	Male	57	Yes	Mother, LC	LADC	IV	R776H	T790M	Osimertinib (yes, 5 m)
18	Male	61	Yes	No	LADC	IV	P848L		Icotinib (yes, 2 m)
									Afatinib (yes, 1 m)
19	Male	80	No	NA	LADC	IV	R831H		Gefitinib (yes, 14 m)

TKI, tyrose kinase inhibitor; NA, not available; LC, lung cancer; BC, breast cancer; LADC, lung adenocarcinoma; EC, esophagus cancer; RC, rectal cancer; LMC, lung mucoepidermoid carcinoma; CT, chemotherapy; DOT, duration of treatment.

## Discussion

In this study, we identified 22 *EGFR* germline mutations in 64 out of 31,906 Chinese patients with lung cancer. The prevalence of *EGFR* germline mutations was 0.2%. The median age at diagnosis in our cohort was similar to that of the general Chinese population, which suggests that new lung cancer cases occur most frequently in individuals aged 60–74 years (1). The proportion of patients with multiple primary cancers in our study was higher than that reported (0.4%–2.4%) in the general Chinese population (16). In addition, the proportion of patients with cancer family history was remarkably high in our cohort. Unfortunately, none of the family members with cancer underwent genetic testing to confirm the presence of the corresponding germline mutations. These findings suggest that genetic susceptibility may play a role in the development of lung cancer. Germline *EGFR* mutations may not contribute to early onset of lung cancer. However, germline mutation analysis should be considered for patients with multiple primary carcinomas or cancer family history.

Our study revealed a unique *EGFR* germline mutation profile in Chinese patients with lung cancer. G863D, the most frequent *EGFR* germline mutation in our cohort, has not been previously reported as a germline mutation. Additionally, R836C, V897A, A647T, V689M, T725M, D761Y, R776S, V765M, V774M, P753S, and K754E were also reported for the first time as germline mutations in this large-scale, retrospective study. Somatic *EGFR* mutation rate in our study was 51.6%, similar to the 50.2% reported by the PIONEER study of Chinese patients with lung adenocarcinoma (17). However, the distribution of *EGFR* somatic mutations differed from that found by another study. In this study, the proportion of L858R, exon 19 deletion, and rare sensitive mutation in patients with somatic *EGFR* mutations was 50%, 17.6%, and 32.4%, respectively. In a previous study, L858R, exon 19 deletion, and other mutations accounted for 40%–45%, 45%, and 10% of *EGFR* mutations, respectively (18). Our study found unique clinical features for patients harboring different germline mutations. D1014N and T790M mutations were common in young patients. The family members of patients with P848L, R776H, V769M, and V774M more commonly suffered from various cancers. The distributions of *EGFR* somatic mutations among patients with different germline mutations were also different. Future studies should confirm whether the unique distribution of *EGFR* somatic mutations may influence the efficacy of EGFR-TKIs in patients with germline *EGFR* mutations.

The response to EGFR-TKIs differed among patients with different somatic and germline *EGFR* mutations. Multiple preclinical studies have suggested that P848L mutation is not a sensitive type (19, 20). The progression-free survival of patients with somatic and germline P848L mutation using erlotinib was 78 days and 4 months, respectively (21, 22). In our study, patients with P848L alone or combined with L858R somatic mutation did not respond to EGFR-TKIs. However, germline P848L combined with exon 19 deletion was sensitive to gefitinib and icotinib. V769M mutation has previously shown controversial and more insensitive efficacy to EGFR-TKIs (23–25), which influenced the effectiveness of EGFR-TKIs in patients with somatic L858R mutations but not in patients with exon 19 deletion mutations. A similar response pattern was observed for patients with K757R mutation, which previously showed more sensitive efficacy to EGFR-TKIs (15, 22). Similar to the previous favorable efficacy of gefitinib in one patient with germline D1014N and somatic L858R mutations (15), two patients in our study also showed good response to EGFR-TKIs. Previous studies suggested that R836C showed inconsistent responses to gefitinib in two cases (26, 27). In our study, modest survival was observed in one patient with germline R836C and somatic exon 19 deletion mutations. In our study, K754E, less sensitive to erlotinib than wild-type EGFR (28), showed modest sensitivity in a patient with concurrent somatic exon 19 deletion mutation. Multiple previous studies have reported the durable response to both first- and third-generation EGFR-TKIs in patients with germline T790M and somatic-sensitive mutations (29–31), which was also observed in our study. Patients with R776H and known sensitive mutations showed sensitivity to EGFR-TKIs such as gefitinib and erlotinib (32, 33). In our study, one patient with germline R776H and somatic T790M mutation showed modest sensitivity to osimertinib. Several studies demonstrated that V774M showed modest sensitivity to EGFR-TKIs (22, 34). In this study, afatinib combined with chemotherapy greatly prolonged survival time for the patient with germline V774M and somatic S768I mutation. R831H was reported to be a ligand-dependent activating mutation with sensitivity to erlotinib (35). One patient with germline R831H mutation responded well to gefitinib treatment.

We recognized several potential limitations in our study. Owing to the low prevalence of *EGFR* germline mutations in lung cancer patients, this study is a retrospective descriptive study. Only two patients (P18 and P19) with *EGFR* germline mutations received EGFR-TKI treatment; hence, we could not evaluate the efficacy difference among patients with (*n* = 17) or without (*n* = 2) *EGFR* somatic mutations. Therefore, we could not determine whether *EGFR* germline mutations should be regarded as driver mutations.

In conclusion, a small number of Chinese patients with lung cancer harbored unique and dispersive *EGFR* germline mutations, which may be related to their second primary carcinomas and cancer family history. Patients with different germline *EGFR* mutations showed unique clinical and genetic characteristics and variant response patterns to EGFR-TKIs treatment.

## Data Availability Statement

The data analyzed in this study is subject to the following licenses/restrictions: This study recruited a total of 31,906 Chinese patients with lung cancer who underwent matched tumor-normal next-generation sequencing (NGS) at Geneplus-Beijing (Beijing, China) between April 2015 and March 2021. Requests to access these datasets should be directed to “Xin Yi, https://www.geneplus.org.cn”.

## Ethics Statement

The studies involving human participants were reviewed and approved by the Ethics Committee of the First Affiliated Hospital, Guangzhou Medical University (Guangzhou, China) (Approval No. 2020-140). The patients/participants provided their written informed consent to participate in this study.

## Author Contributions

XL, MC, and CZ designed the study. MP wrote and edited the manuscript, which was approved by all the authors. QC, HD, and JD evaluated and examined the data. OL and YW performed the data collection. MY and RC analyzed the data. All authors contributed to the article and approved the submitted version.

## Funding

This study was supported by the Natural Science Foundation of Changsha (No. kq2014001); State Key Laboratory of Respiratory Disease-The open project (SKLRD-OP-202111); Zhongnanshan Medical Foundation of Guangdong Province (ZNSA-2020003); Fundamental and Applied Fundamental Research Project of City-School (Institute) Joint Funding Project, Guangzhou Science and Technology Bureau (202102010357); and Wu Jieping Medical Foundation (320.6750.2020-19-8).

## Conflict of Interest

Authors MY and RC were employed by Geneplus-Beijing.

The remaining authors declare that the research was conducted in the absence of any commercial or financial relationships that could be construed as a potential conflict of interest.

## Publisher’s Note

All claims expressed in this article are solely those of the authors and do not necessarily represent those of their affiliated organizations, or those of the publisher, the editors and the reviewers. Any product that may be evaluated in this article, or claim that may be made by its manufacturer, is not guaranteed or endorsed by the publisher.
